# A Bill for the Prevention of Premature Burial

**Published:** 1907-12-07

**Authors:** 


### A Bill for the Prevention of Premature Burial.

A draft of a measure promoted by the Association for the Prevention of Premature Burial has been submitted to us. It proposes a number of much-needed changes in the law relating to death certification, the more important of which have been demanded by the medical profession in this country for over 30 years. That it should have been left to the twentieth century to enact that before a person be legally buried he should first be certified by a competent person to be dead illustrates in what strange fashion the law lags behind the most rudimentary requirements of common sense. As our readers are well aware, the state of the law in relation to death certification and burial is such that a person may die and be buried without being seen either alive or dead by a medical man, and over 8,000 persons were buried last year without the cause of death being certified by a registered medical practitioner. The fact of death need never be certified, and probably the greater number of bodies are interred without this significant preliminary to legal burial. There is nothing in the law requiring that the person buried shall be known to be the person to whom the certificate relates, and, in fact, certificates of the cause of death may be granted in the existing state of the law in respect of persons who do not die, and funerals may take place in which such persons are or are not buried.

The Bill before us, in the event of its becoming law in its present form, would amply safeguard against the contingency which it is designed to prevent, namely, premature burial. The Bill requires that "Every certificate of the cause of death shall, in addition to stating the cause of death, also state the signs from which the registered medical practitioner giving such certificate infers the fact of death, and no medical practitioner shall sign any such certificate unless he has previously inspected the body of the deceased person. It provides for the granting of certificates of the fact of death where the cause cannot be certified, and makes it a misdemeanour for any person to bury, cremate, embalm, dissect, or perform a funeral service in the absence of a death certificate in respect of the deceased. The Bill is drafted with a view to prevent premature burial, a dire but extremely remote contingency. Incidentally, with a view to this end, it removes some of the most glaring defects in the law as it exists. In addition, it has the merit, frequently lacking in measures affecting the medical profession, that it proposes that professional services shall be remunerated.

But the Bill has the defects of its somewhat narrow purpose. It is not such a reform of the Births and Deaths Registration Act as is called for upon broader considerations than those which its promoters contemplate. Infinitely more serious than the horrible, rare, but gruesomely fascinating contingency which seizes the fearful imaginations of those who live in dread of being buried alive, are the actual dangers which result from our defective system of death certification. When a clumsy poisoner blunders into unavoidable detection by his crass stupidity, it is usually found that numerous victims of his dull-witted crimes have been quite respectably buried as being presumably naturally dead. But even the medico-legal aspects of the position are insignificant beside the public health concern in the causes of mortality. For, after all, a small minority of people die from overt criminal acts, while a large majority die from causes which a more rational ordering of life would prevent. The registration and further investigation of the causes of death should be a province of public health administration. The Medical Officer of Health should not be the recipient of *copies* of certificates of death, for which the local authorities pay the Registrars of Deaths, notwithstanding that the medical practitioners who grant the originals are unpaid. Death registration, investigation and verification, we say, should be provinces of the public health departments.

Far-reaching changes of this kind cannot be dealt with in a private Bill, and the need for such changes is even accentuated by the measure before us. At the least the Bill will serve as another reminder of the need for radical changes in the law relating to death certification. Several of the clauses are drafted so as to leave little to be desired, but since the problem is wider than is contemplated by the promoters of the Bill, we hope that it will accomplish its purpose by stimulating the legislature to deal comprehensively with this urgent question.

